# Evaluation of a broad-ranging and convenient enzyme-linked immunosorbent assay using the lysate of infected cells with five serotypes of *Orientia tsutsugamushi*, a causative agent of scrub typhus

**DOI:** 10.1186/s12866-016-0910-5

**Published:** 2017-01-05

**Authors:** Motohiko Ogawa, Masaaki Satoh, Masayuki Saijo, Shuji Ando

**Affiliations:** Department of Virology 1, National Institute of Infectious Diseases, 1-23-1, Toyama, Shinjuku-ku, Tokyo, 162-8640 Japan

**Keywords:** ELISA, Serological test, Scrub typhus, *O. tsutsugamushi*

## Abstract

**Background:**

Scrub typhus is a mite-borne rickettsiosis caused by infection of *Orientia tsutsugamushi*, which is endemic to several Asia-Pacific Rim countries, including Japan. Although micro-indirect immunofluorescent assay (micro-IFA) is the standard method for the serological diagnosis of scrub typhus, enzyme-linked immunosorbent assay (ELISA) is considered to be more objective, by providing digitized results as opposed to being subject to the judgment of the evaluator as in micro-IFA. Therefore, the aim of this study was to develop a broad-ranging ELISA using the five major prevalent serotypes of *O. tsutsugamushi* in Japan as the antigens. Furthermore, in contrast to previous studies that used purified microorganisms via ultracentrifugation, we directly used the infected cells, and evaluated the diagnostic accuracy of this simplified method to that of micro-IFA.

**Results:**

Evaluation of paired patient sera against the five serotypes showed that the accuracy of ELISA relative to micro-IFA was 87.4 and 79.5% for immunoglobulin (Ig)M and IgG assays, respectively, at the optimized cut-off value. Further evaluation of patient sera against the expected serotype of the infecting strain showed that the accuracy of ELISA compared to micro-IFA increased to 100 and 97.4% in the IgM and IgG assays, respectively. This suggests that use of the five prevalent serotypes contributed to the increase of the accuracy of ELISA. When applying the criteria of serological diagnosis for paired sera samples to ELISA, all 19 patients were diagnosed as positive; a ≥4-fold elevation of the antibody titer was observed in 15 of 19 patients that were positive, and very high antibody titers were observed in both paired sera samples of the remaining four patients. In addition, all samples of healthy subjects and patients with other types of rickettsiosis were diagnosed as negative using these criteria.

**Conclusions:**

Our results suggest the excellent performance of the new broad-ranging and convenient ELISA, which appears to be applicable for the diagnosis of scrub typhus patients infected with the wide variety of prevalent strains in Japan. Furthermore, the ELISA is more objective than the micro-IFA, and can therefore provide more accurate diagnoses in Japan.

**Electronic supplementary material:**

The online version of this article (doi:10.1186/s12866-016-0910-5) contains supplementary material, which is available to authorized users.

## Background

Scrub typhus is a mite-borne rickettsiosis caused by the intracellular bacterium *Orientia tsutsugamushi* [1]. The microorganism is transmitted to humans through bites from an infected trombiculid mite. Scrub typhus is widely found in several countries of Asian Pacific areas, including Japan [2], where approximately 300–500 cases are reported annually, including a few fatal cases [3].

Serological diagnosis is the main method for confirmation of scrub typhus, because direct detection of the microorganisms is a generally difficult and cumbersome process. For example, isolation of microorganisms requires biosafety level-3 facilities and is a lengthy procedure, and bacterial DNA detection from the blood using polymerase chain reaction (PCR)-based methods is unreliable, as negative results are common, especially once antibiotic treatments have been initiated. Recently, DNA detection from the eschar was shown to be useful [4]; however, the eschar is absent in some cases. Furthermore, this procedure requires a skin biopsy, and is therefore very invasive to the patients, which has been the main obstacle to its widespread use. For the serological diagnosis of scrub typhus, it is recommended to use at least five serotypes of *O. tsutsugamushi* in Japan, such as the Kato, Karp, Gilliam, Kuroki [5], and Kawasaki [6] types, which are the most prevalent serotypes in Japan and generally show limited serological cross-reactivity in patient sera [7]. That is, the serum antibody level of the patient will only be elevated against a specific serotype and not in response to the other serotypes. These facts clearly suggest that five or more strains should be used in serological tests of scrub typhus in Japan, although some previous reports from other countries used only a few standard strains in serological tests [8, 9].

The micro-indirect immunofluorescent assay (micro-IFA) [10, 11] is currently considered the gold-standard method for the serological diagnosis of scrub typhus worldwide. In Japan, the micro-IFA procedure involves spotting infected cells of the five serotypes mentioned above in one well of a multi-well slide. Therefore, the five antigens can be simultaneously observed in one well requiring only a small volume of serum for a given assay. This multi-well slide is a widely used antigen slide in Japan, and is employed in certain national and regional public health laboratories for the serological diagnosis of scrub typhus.

However, micro-IFA is associated with a major disadvantage, in that the end point of interpretation of the antibody titer can vary for the same sample depending on the evaluation of the individual conducting of the serological test according to their ability and/or experience [12, 13]. Therefore, the judgment of micro-IFA results is heavily subjective. In particular, the end point can be variable when the fluorescent background is high, which can lead to false-positive results of serological diagnosis. As an alternative, an enzyme-linked immunosorbent assay (ELISA) can serve as a more objective method than micro-IFA for serological diagnosis, mainly because digitized results are obtained.

In this study, we developed a broad-ranging and simplified ELISA directly using infected cells of the five major prevalent serotypes of *O. tsutsugamushi* in Japan. In addition to the objectivity advantage, this method has additional convenience, since previous methods for ELISA for rickettsia have involved a purification step of the sample by ultracentrifugation as the antigen [8, 9, 14]. We evaluated the accuracy of this novel broad-ranging and simplified ELISA in comparison with the micro-IFA for the serological diagnosis of scrub typhus in Japan.

## Methods

### Preparation of the antigen plate for ELISA

Five different serotypes of *O. tsutsugamushi*, Kato, Karp, Gilliam, Kuroki, and Kawasaki type, were used. Kato, Karp, and Gilliam are international standard strains, whereas Kuroki and Kawasaki are unique isolates in Japan. Diversity of the five strains that was based on the amino acid sequences of the type specific antigens (56 kDa major outer membrane proteins) was shown in Additional file 1. All the strains are routinely maintained in our laboratory. The mouse fibroblast cell line L-929 (JCRB9003), purchased from JCRN cell bank, National Institute of Biomedical Innovation, Japan, was infected with the strain of each serotype [15], and the cells were harvested once the cytopathic effect was apparent. Infected cells were collected by spinning at 1800 rpm (1,000 g) at 4 °C and were lysed in phosphate buffered saline (PBS) containing 2% Triton-X114 at 4 °C (approximately 1 × 10^7^ cells from a 75-cm^2^ flask/ml). The cell lysate was mixed well by vortexing, and then the supernatant was collected after spinning at 10,000 rpm (9,100 g) at 4 °C. The numbers of microorganisms in each cell lysate were quantified by real-time PCR as previously described [15]. According to the quantification, the cell lysate of the infected cells was diluted with PBS at a concentration of approximately 2000 microorganisms/ml. One hundred microliters of the diluted lysate was added to the wells of a 96-well microwell plate and incubated for 18 h at 4 °C. The placement of each lysate on the plate is shown in Additional file 2. After washing with 200 μl of PBS containing 0.5% Tween 20 (PBS-T) three times, 100 μl of PBS-T containing 5% skim milk (*w/v*) (PBS-TM) was added to each well, and the plate was incubated for 4 h at room temperature for blocking. After washing with 200 μl of PBS-T three more times, the antigen plate was stored at −80 °C until use.

### Human serum samples

Nineteen paired sera samples of scrub typhus patients (*N* = 38) and 18 paired sera samples of healthy subjects (*N* = 36) were used as the positive and negative sera samples, respectively, to determine the cut-off value of ELISA. In addition, 12 paired sera samples of patients with other types of rickettsiosis, including 9 pairs of sera samples of patients with Japanese spotted fever (*N* = 18) and three pairs of sera samples of patients with murine typhus (*N* = 6) were used to evaluate the specificity of ELISA. These serum samples were collected from 2000 to 2012 and sent to our laboratory for serological tests of scrub typhus and other rickettsiosis. This study was approved by the Ethics Committee for Medical and Health Research involving Human Subjects of the National Institute of Infectious Disease (474: 16th Jan, 2014).

### ELISA

The serum was diluted 4-fold serially from 1:100 to 1:6400 with PBS-TM, and 100 μl of each diluted serum sample was applied to two wells (for IgM and IgG) of each serotype of *O. tsutsugamushi* and mock (the lysate of non-infected cells) (see Additional file 2). The sera were incubated for 2 h at room temperature and then washed with 200 μl of PBS-T three times. Subsequently, horseradish peroxidase (HRP)-conjugated AffiniPure goat anti-human IgM, Fc5μ fragment-specific or IgG Fcγ antibodies (Jackson ImmunoResearch Laboratories Inc., West Grove, PA, USA) were diluted at 1:10,000 with PBS-TM according to the manufacturer instructions, and 100 μl of each diluted HRP-conjugated antibody was applied to one of the two wells incubated with the same serum (see Additional file 2). After incubating for 1 h at room temperature, the plate was washed four times with 200 μl of PBS-T. Finally, 100 μl of 2,2′-azino-bis(3-ethylbenzothiazoline-6-sulfonic acid) (ABTS) solution (Roche Diagnostics, Mannheim, Germany) was added to each well and incubated for 30 min at room temperature; 50 μl of stop solution (0.1 N oxalic acid) was added to terminate the reaction. The optical density at 405 nm (OD_405_) of each well was determined by measuring absorbance in a spectrophotometer (Multiskan Ascent, Thermo Fisher Scientific, Waltham, MA USA). The ELISA value of each serum sample was calculated by subtracting the OD_405_ against the mock well from the OD_405_ against each serotype.

### Micro-IFA

A previously reported micro-IFA method [9] was modified and applied for determination of the antibody titer of each serum sample. In brief, the infected cells of the five serotypes were spotted in each well of a multi-well slide. The serum was diluted two-fold serially from an initial 1:40 dilution with PBS. After the diluted serum was added to one well, the slides were incubated at 37 °C for 1.5 h in a humid chamber. After washing with PBS-T, Alexa Fluor 488-conjugated AffiniPure goat anti-human IgM, Fc5μ or IgG Fcγ fragment-specific antibodies (Jackson ImmunoResearch Laboratories Inc., West Grove, PA, USA) were diluted at 1:200 with PBS containing 0.01% evans blue and applied onto the well. The slides were then incubated at 37 °C for 1.5 h. After washing with PBS-T, the slides were embedded with a mounting fluid (Mount, PermaFluor, Thermo Fisher Scientific, Waltham, MA, USA), topped with a micro-cover glass, and observed under a fluorescent microscope (Axioskop2 Plus; Carl Zeiss, Oberkochen, Germany). The micro-IFA titer was calculated as the highest dilution of serum at which the fluorescent-stained typical rickettsial morphology was observed. The micro-IFA titer was determined in two or three independent observations.

### Data analysis

The mean ELISA value + 2, 3, and 4 standard deviations (SD) of the 18 pairs of sera from healthy subjects were applied as cut-off values to find the most suitable value, whereas for micro-IFA, the common cut off value of ≥1:80 that is routinely used in Japan was applied. Serological diagnosis was judged using the temporal criteria of ELISA of an IgM value > the mean + 3 SD for a single serum sample and/or ≥4-fold elevation of ELISA antibody titers for the paired sera (by comparison of ELISA values greater than the mean + 3 SD among those diluted at 1:100, 1:400, 1:1,600, and 1:6,400). Micro-IFA results were judged according to the standard criteria of IgM ≥1:80 for a single serum sample and/or ≥4-fold elevation of micro-IFA titers for paired sera. The results of ELISA and micro-IFA were compared with non-linear regression analysis. All statistical analyses were conducted with Excel software (Microsoft Corporation, WA, USA).

## Results

### Evaluation of the cut-off value of ELISA against the five serotypes

**Fig. 1:**
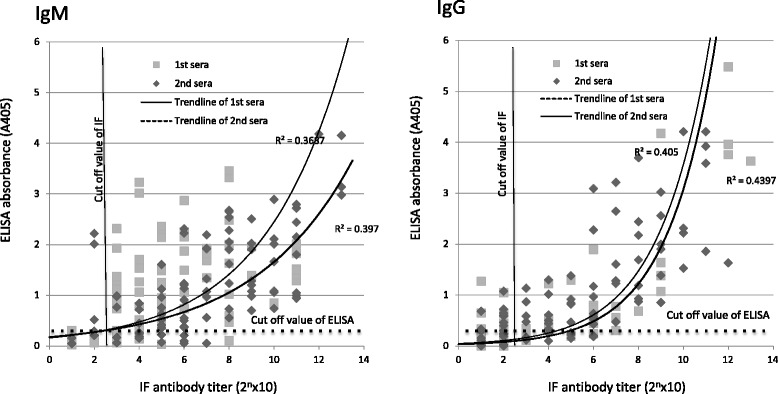
Evaluation of the cut-off value of ELISA for scrub typhus. Scattergram of (**a**) IgM and **b** IgG ELISA and the micro-IFA reactivity of sera pairs from 19 patients against five serotypes of *O. tsutsugamushi* strains. Each square and diamond shows the reactivity of the first and second serum samples against each antigen, respectively. The solid line shows the cut-off value of micro-IFA (1:80) that is generally used in Japan. The dotted line shows the candidate cut-off line of ELISA (the mean + n SD of 18 sera pairs of healthy subjects). Non-linear regression analysis was performed showing the correlations (R^2^ values) between ELISA results and micro-IFA titers

To evaluate the performance of the ELISA developed in this study, the ELISA value at the serum dilution of 1:100 was compared with the determined micro-IFA titer (Fig. 1). In the non-linear regression analysis, moderate correlations were found between ELISA values and micro-IFA titers in IgG assays, whereas weak correlations were found in IgM assays.

In the evaluation of cut off-values of ELISA, the mean (SD) values of the healthy sera for the IgM and IgG assays were 0.0010 (0.0593) and 0.0279 (0.0614), respectively. The ELISA values and micro-IFA titers of the 19 paired sera samples of scrub typhus patients against the five serotypes, and their concordance are presented in Fig. 1. In the IgM assay, positive conformity rates between the ELISA and micro-IFA were 78.9, 77.4, and 75.3%, whereas negative conformity rates were 7.4, 10.0, and 10.5%, when applying the cut-off values for +2, 3, and 4 SD of the mean, respectively. Total conformity rates between ELISA and micro-IFA in the IgM assay were 86.3, 87.4 and 85.8% with each cut-off value, respectively. In the IgG assay, positive conformity rates between the ELISA and micro-IFA were 50.5, 47.4, and 43.2%, whereas negative conformity rates were 26.3, 32.1, and 35.3% using the cut-off values of + 2, 3, and 4 SD of the mean, respectively. Total conformity rates between ELISA and micro-IFA in the IgG assay were 76.8, 79.5, and 78.5% with each cut-off value, respectively. In the assay of both IgM and IgG, the results showed the highest total conformity with the mean + 3 SD cut-off value. However, there were discrepancies in 13.6 and 20.5% of the results between ELISA and micro-IFA in the IgM and IgG assays, respectively, when applying this cut-off value.

### Evaluation of ELISA against the expected serotype of the infecting strain

**Fig. 2:**
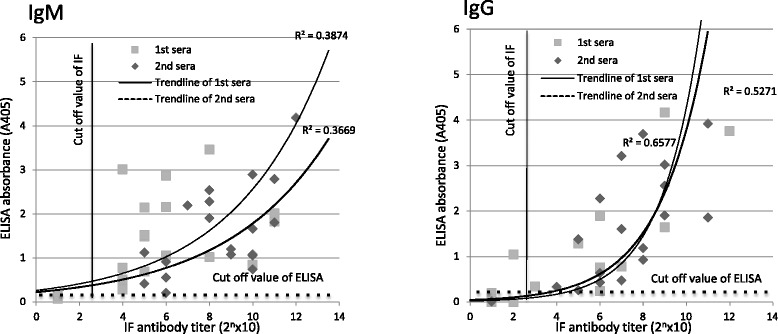
ELISA and micro-IFA using only the expected serotype of *O. tsutsugamushi* strains in patient sera. The expected serotype of the infecting strain was determined as the most reactive serotype showing the highest ELISA value. Paired sera from 19 patients were used. Each square and diamond shows the reactivity of the first and the second serum sample against the expected serotype of the infecting strain, respectively. The cut-off values of the mean + 3 SD and 1:80 were adopted for ELISA and micro-IFA, respectively. Non-linear regression analysis was performed to determine correlations (R^2^) between the ELISA results and micro-IFA titers

The expected serotype of the strain that infected the patients in this study was defined as the serotype against which the sera of each patient showed the highest titer. ELISA values and micro-IFA titers of the 19 paired serum samples of scrub typhus patients against only the expected infecting strain are shown in Fig. 2. In the non-linear regression analysis, moderate correlations were found between ELISA values and micro-IFA titers in the IgG assays, whereas weak correlations were found in the IgM assays. Next, the mean + 3 SD and ≥1:80 cut-off values were applied for ELISA and micro-IFA, respectively, for comparison to the previous analysis described above with all serotypes. In the IgM and IgG assays, positive conformity rates between ELISA and micro-IFA were 94.7 and 71.1%, whereas negative conformity rates were 5.3 and 26.3%, respectively. Total conformity rates between ELISA and micro-IFA were 100 and 97.4%, respectively.

### Specificity of ELISA

**Fig. 3:**
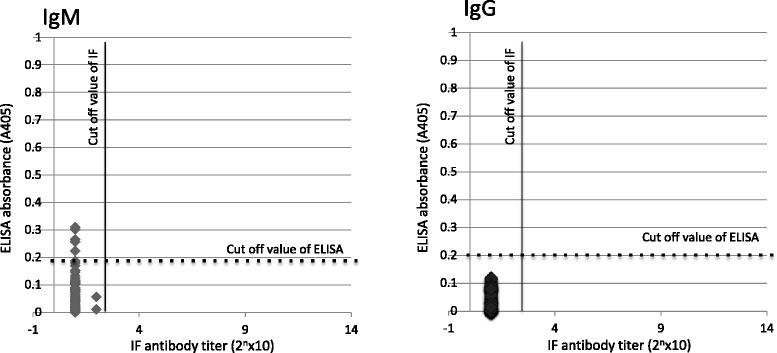
Specificity of IgM and IgG ELISA for scrub typhus. Specificity was examined by testing sera pairs of 12 patients with other types of rickettsiosis, including 9 patients with Japanese spotted fever and 3 patients with murine typhus

The specificity of ELISA was examined by comparing the ELISA values and micro-IFA titers from paired serum samples of Japanese patients with other types of rickettsiosis, and the results are shown in Fig. 3. All sera were negative in both the IgM and IgG micro-IFA at the cut-off value of ≥1:80. In the IgM ELISA, the second serum sample of one patient with Japanese spotted fever and the first serum sample of one patient with murine typhus were slightly positive against all five strains and two of the five strains respectively. Overall, only 7 results (5.8%) were slightly positive, whereas in the IgG assay, no sera were positive at the cut-off value of the mean + 3 SD.

### Comparison of serological diagnosis by the temporal criteria of ELISA and the standard criteria of micro-IFA

The serological diagnoses of the 19 patients according to the above-mentioned criteria are shown in Table 1. Using the criteria for a single serum sample for the first serum sample, 18 patients were positive in ELISA, whereas 17 patients were positive in micro-IFA. Using the criteria for the paired sera, 15 patients were positive and 4 patients were negative in both ELISA and micro-IFA, and there were discrepancies between the results of the assays for two patients. One patient determined to be negative with ELISA and two patients determined to be negative with micro-IFA according to the criteria of a single serum sample were found to be positive using the criteria for paired sera. Four patients determined to be negative in both ELISA and micro-IFA according to the criteria of paired sera were apparently positive based on the criteria of single serum samples; this was attributed to the lack of detection of the very high increase (≥4-fold) of antibody titer between the paired sera. In these patients, the IgM antibody titers of the first and second sera were both very high. Finally, using both criteria, all 19 patients were serologically diagnosed as positive in ELISA as well in micro-IFA.

**Table 1:** Comparison of serological diagnosis by the novel criteria of ELISA and the stanndard criteria of IF assay

Patients		No1	No2	No3	No4	No5	No6	No7	No8	No9	No10	No11	No12	No13	No14	No15	No16	No17	No18	No19
ELISA	IgM > Av + 3SD 1)	+	+	+	+	+	+	+	+	+	+	-	+	+	+	+	+	+	+	+
	Eelevation of antibody between paired sera 2)	+	+	+	+	+	+	-	+	+	+	+	-	+	+	-	+	-	+	+
	Diagonosis	P 3)	P	P	P	P	P	P	P	P	P	P	P	P	P	P	P	P	P	P
IF	IgM ≥80	+	+	+	+	+	+	+	+	+	+	-	+	+	-	+	+	+	+	+
	Eelevation of antibody between paired sera	+	+	+	+	+	+	-	+	-	+	+	-	+	+	-	+	+	+	+
	Diagonosis	P	P	P	P	P	P	P	P	P	P	P	P	P	P	P	P	P	P	P

1) IgM antibody elevation in acutephase sera, 2) ≥4 or more folds-elevation of antibody titer between paired sera, 3) P:positive

Furthermore, the second serum samples of one healthy subject and one patient with Japanese spotted fever, and the first serum sample of a patient with murine typhus were positive according to the criteria of a single serum sample. Their IgM ELISA values were very close to the cut-off value, although these cases were all diagnosed as negative according to the criteria of paired sera (data not shown).

## Discussion

In Japan, micro-IFA is the standard serological diagnostic method for scrub typhus. However, the interpretation of this assay is subjective and requires a highly trained person to accurately determine the precise end point. Therefore, the end point for the same sample can be discordant depending on the individual conducting the test, especially when the fluorescent background is high. This fact sometimes leads to false-positive results in serological diagnosis. In addition, the antigen slide used for micro-IFA is generally hand-made with a pen cap [10, 11], and therefore also requires a highly trained and skilled technician to prepare stable and high-quality antigen slides to avoid variable results among the slides. The use of ELISA can compensate for these disadvantages of the micro-IFA, mainly because the results are digitized and are thus more objective, and antigen plates with a stable quality can be easily prepared.

One of the most important factors for establishment of a serological diagnostic method is to confirm the match between the antigen used and the expected infecting strain. In the case of scrub typhus, our previous report showed that the serum antibody level of some patients was only elevated against limited serotypes, whereas that of most patients was elevated against all serotypes [7]. In some previous studies conducted outside of Japan, serological diagnostic methods such as ELISA were evaluated using only certain reference serotypes that did not include some of the most prevalent serotypes in Japan [8, 9], which can show wider variation than those of other countries. Accordingly, the sera from patients infected with a different serotype in Japan might not react to the serotypes used in these methods tested in previous reports. Therefore, to achieve high sensitivity of a serological test for scrub typhus, it is strongly recommended that the most prevalent serotypes in each region should be used as much as possible. At least five serotypes should be used for the diagnosis of scrub typhoid in Japan. Given the above background, in this study, a broad-ranging ELISA that covered the prevalent five major serotypes of *O. tsutsugamushi* in Japan (Kato, Karp, Gilliam, Kuroki, and Kawasaki) was established. The digitized ELISA value was obtained by subtracting the absorbance of the mock well (lysate of non-infected cells) from the absorbance of the well with each serotype. Therefore, even when the background is high, only the specific absorbance of each serotype can be effectively evaluated with this method. For evaluation of the ELISA cut-off value, the reaction of each serum sample against the strains comprising the five serotypes was examined. Even when using the optimized cut-off value, the mean + 3 SD, some discrepancies in the results of ELISA and micro-IFA were observed. These discrepancies were most likely due to differences in the extent of serological cross-reactions against the five serotypes among patients. These differences can be caused by variations in the serotype of the infecting strain and individual immune reactions, as well as the different nature of the adopted antigens, since the infected cells themselves were used for micro-IFA, whereas the lysates of the infected cells were used for ELISA. This suggests that the conformational epitope of rickettsia may be slightly but not drastically changed in the lysate of infected cells.

To solve these discrepancies, we also evaluated the results against only the expected serotype of the infecting strain, which was that showing the highest ELISA value in each patient. Non-linear regression analysis showed that the correlations between ELISA values and micro-IFA titers were higher when using the expected infecting serotype compared to those obtained against all five serotypes in the IgG assays, whereas the correlations were similar with the IgM assays. Furthermore, using the optimized cut-off value, the specificity and sensitivity of the ELISA results were relatively consistent with those of the micro-IFA results for both the IgM and IgG assays. These findings strongly suggest that the performance of ELISA was as high as that of micro-IFA. These results also suggested that the serotype that is most similar to the actual infecting strain should be used to obtain high specificity and sensitivity, and that adoption of the most prevalent plural serotypes for antigens is important to obtain the best serotype match between the antigen and infecting strain.

In general, for the criteria of serological diagnosis, it is recommended that an elevation of ≥4-fold between paired sera should be observed, especially when the antibody titer is close to the cut-off value used as the criterion for a single serum sample. As similar criteria used for micro-IFA were applied to ELISA, all 19 of the scrub typhus patients were diagnosed as positive. Furthermore, all of the healthy subjects and patients with other types of rickettsiosis reported in Japan [3, 16–18] were diagnosed as negative according to the criteria. Therefore, these results demonstrate that the sensitivity and specificity of the ELISA developed in this study by the applied criteria are practical for use in Japan.

Another advantage of this study is that cells infected with *O. tsutsugamushi* were used, which is in contrast to previous studies that generally used purified microorganisms as the rickettsial antigens [9, 19]. An ultracentrifugation step is required for the purification of microorganisms, which requires expensive equipment and generally more time. In this study, the same infected cells that were used for the preparation of the antigen in the micro-IFA were used to prepare the lysate of the antigens for ELISA. Therefore, preparation of the antigen lysate and plate was very easy and could be readily applied in most laboratories in Japan.

## Conclusion

In this study, we established a broad-ranging and convenient ELISA for the diagnosis of scrub typhus in Japan using five different serotypes of antigens, which appears to be suitable for the diagnosis of scrub typhus patients infected with the wide variety of strains circulating in Japan. The specificity, sensitivity, and practicality of the broad-ranging ELISA were as excellent as the gold-standard serological diagnostic method, micro-IFA. In addition, judgment of the results in ELISA is more objective than that of IFA, mainly because a homogeneous and stable quality of the antigen is coated on the plates and digitized results are obtained in ELISA. These facts clearly suggest that the ELISA developed in this study will be more accurate than micro-IFA for the serological diagnosis of scrub typhus in Japan. Furthermore, the ELISA has a great advantage for serological screening in general epidemiological research owing to its ability to handle several sera samples simultaneously.

## Additional files

Additional file 1: Diversity between the five major strains of *Orientia tsutsugamushi* by phylogenetic analysis based on the amino acid sequences of the type specific antigens. (PPTX 61 kb)
Additional file 2: Placement of antigen, serum and HRP-conjugated antibodies on ELISA plate in this study. (PPTX 82 kb)
Additional file 3: The raw data of ELISA and micro-IF generated and analyzed during this study. (PDF 110 kb)
