# Supplementary material for: Evaluation of a broad-ranging and convenient enzyme-linked immunosorbent assay using the lysate of infected cells with five serotypes of Orientia tsutsugamushi, a causative agent of scrub typhus
Source: BMC Microbiol. 2017 Jan 5;17:7. doi: 10.1186/s12866-016-0910-5 (PMC5217197; doi:10.1186/s12866-016-0910-5)
Supplement: Additional file 1: — Diversity between the five major strains of Orientia tsutsugamushi by phylogenetic analysis based on the amino acid sequences of the type specific antigens. (PPTX 61 kb) [file 12866_2016_910_MOESM1_ESM.pptx]

## Slide 1
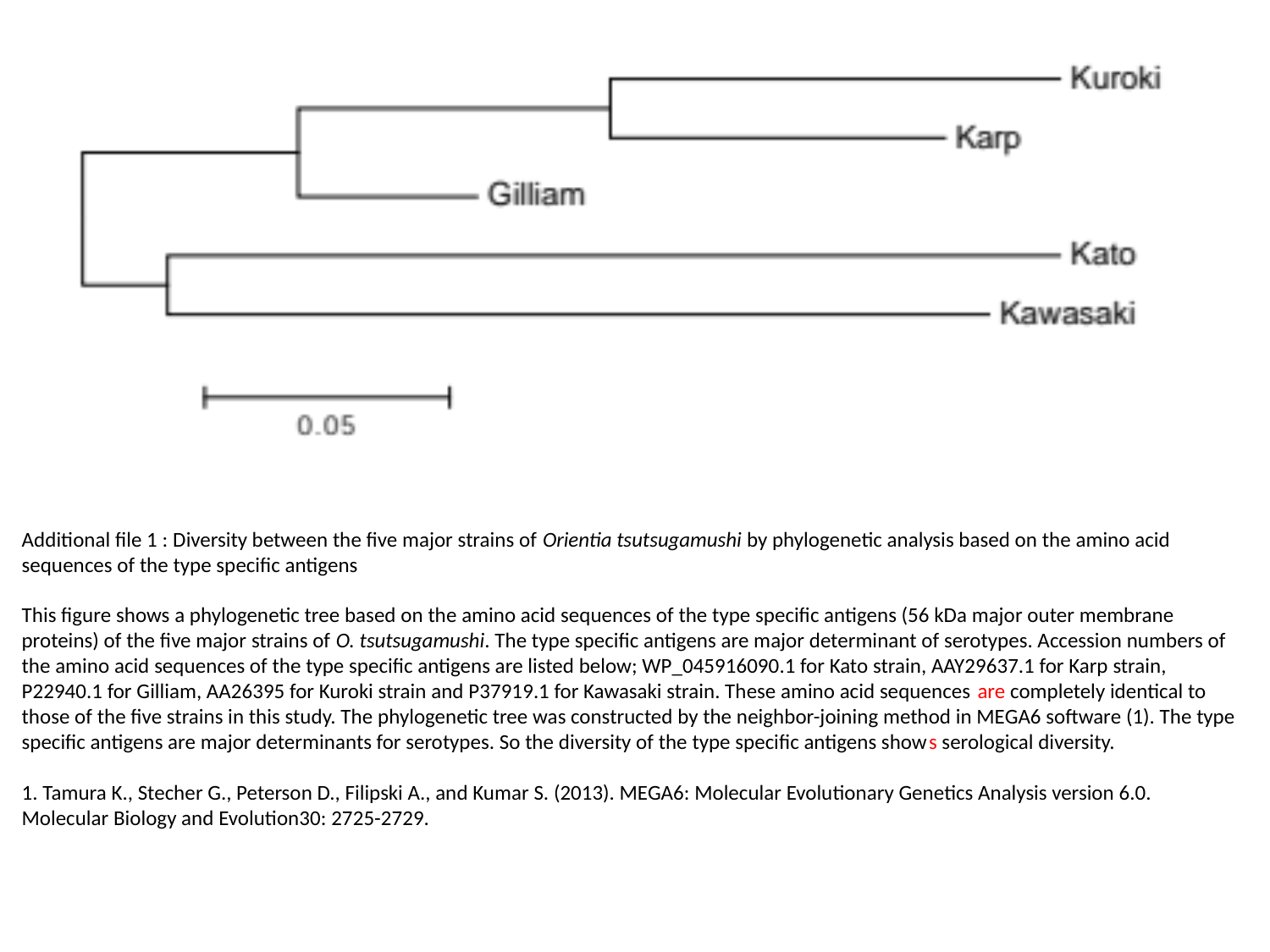

Additional file 1 : Diversity between the five major strains of Orientia tsutsugamushi by phylogenetic analysis based on the amino acid sequences of the type specific antigens
This figure shows a phylogenetic tree based on the amino acid sequences of the type specific antigens (56 kDa major outer membrane proteins) of the five major strains of O. tsutsugamushi. The type specific antigens are major determinant of serotypes. Accession numbers of the amino acid sequences of the type specific antigens are listed below; WP_045916090.1 for Kato strain, AAY29637.1 for Karp strain, P22940.1 for Gilliam, AA26395 for Kuroki strain and P37919.1 for Kawasaki strain. These amino acid sequences are completely identical to those of the five strains in this study. The phylogenetic tree was constructed by the neighbor-joining method in MEGA6 software (1). The type specific antigens are major determinants for serotypes. So the diversity of the type specific antigens shows serological diversity.
1. Tamura K., Stecher G., Peterson D., Filipski A., and Kumar S. (2013). MEGA6: Molecular Evolutionary Genetics Analysis version 6.0. Molecular Biology and Evolution30: 2725-2729.
