# Supplementary material for: Evaluation of a broad-ranging and convenient enzyme-linked immunosorbent assay using the lysate of infected cells with five serotypes of Orientia tsutsugamushi, a causative agent of scrub typhus
Source: BMC Microbiol. 2017 Jan 5;17:7. doi: 10.1186/s12866-016-0910-5 (PMC5217197; doi:10.1186/s12866-016-0910-5)
Supplement: Additional file 2: — Placement of antigen, serum and HRP-conjugated antibodies on ELISA plate in this study. (PPTX 82 kb) [file 12866_2016_910_MOESM2_ESM.pptx]

## Slide 1
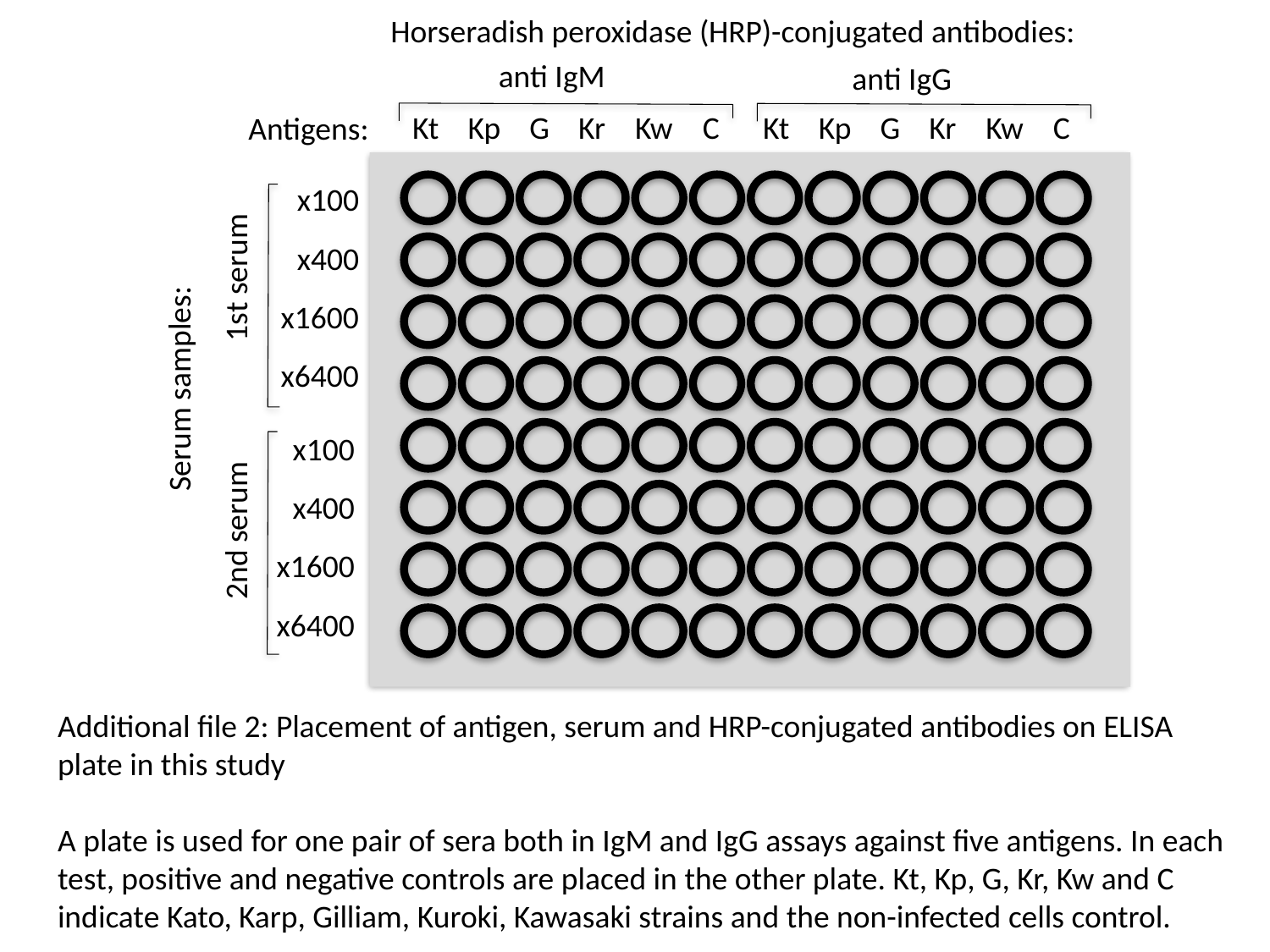

Horseradish peroxidase (HRP)-conjugated antibodies:
anti IgM
anti IgG
Kt
Kp
G
Kr
Kw
C
Kt
Kp
G
Kr
Kw
C
Antigens:
x100
x400
1st serum
x1600
x6400
Serum samples:
x100
x400
2nd serum
x1600
x6400
Additional file 2: Placement of antigen, serum and HRP-conjugated antibodies on ELISA plate in this study
A plate is used for one pair of sera both in IgM and IgG assays against five antigens. In each test, positive and negative controls are placed in the other plate. Kt, Kp, G, Kr, Kw and C indicate Kato, Karp, Gilliam, Kuroki, Kawasaki strains and the non-infected cells control.
