# Supplementary material for: Evaluation of a broad-ranging and convenient enzyme-linked immunosorbent assay using the lysate of infected cells with five serotypes of Orientia tsutsugamushi, a causative agent of scrub typhus
Source: BMC Microbiol. 2017 Jan 5;17:7. doi: 10.1186/s12866-016-0910-5 (PMC5217197; doi:10.1186/s12866-016-0910-5)
Supplement: Additional file 3: — The raw data of ELISA and micro-IF generated and analyzed during this study. (PDF 110 kb) [file 12866_2016_910_MOESM3_ESM.pdf]

The raw data generated and analysed druing this study

1. Sera of scrub typhus patients

Serum No*	Serotype of antigens$	Serum antibody titer - IgM					Serum antibody titer - IgG				
		Micro IF titer	ELISA value				Micro IF titer	ELISA value			
			x100	x400	x1600	x6400		x100	x400	x1600	x6400
OTS-1A	Kt	320	1.254	0.951	0.382	0.142	20	0.078	0.029	0.015	0.01
OTS-1A	Kp	320	1.489	1.024	0.382	0.124	40	0.02	0.005	0.003	0.009
OTS-1A	G	640	2.868	1.612	0.563	0.166	20	0.098	0.029	0.011	0.008
OTS-1A	Kr	640	0.737	0.28	0.105	0.037	20	0.24	0.08	0.021	0.011
OTS-1A	Kw	5120	1.393	0.978	0.446	0.193	20	0.118	0.059	0.025	0.012
OTS-1C	Kt	640	1.215	0.912	0.315	0.1	20	0.159	0.041	0.019	0.005
OTS-1C	Kp	1280	1.324	0.888	0.349	0.106	80	0.131	0.037	0.014	-0.001
OTS-1C	G	2560	2.54	1.435	0.505	0.162	160	0.336	0.157	0.058	0.016
OTS-1C	Kr	1280	0.56	0.323	0.139	0.055	20	0.333	0.123	0.041	0.008
OTS-1C	Kw	5120	1.283	0.895	0.456	0.258	1280	0.472	0.232	0.118	0.037
OTS-2A	Kt	20	0.084	0.037	0.014	0.006	20	0.119	0.014	-0.003	0
OTS-2A	Kp	160	0.141	0.022	0.004	0.001	40	0.158	0.024	-0.001	0
OTS-2A	G	160	0.279	0.07	0.027	0.008	80	0.342	0.115	0.014	0.005
OTS-2A	Kr	40	0.074	0.011	0.002	-0.001	40	0.167	0.051	0.007	0.001
OTS-2A	Kw	40	0.152	0.046	0.035	0.027	40	0.094	0.023	-0.002	-0.002
OTS-2C	Kt	40	0.21	0.059	0.029	0.027	80	0.476	0.127	-0.007	-0.007
OTS-2C	Kp	320	0.072	0.016	0.011	-0.016	160	0.506	0.138	-0.005	-0.025
OTS-2C	G	640	0.209	0.099	0.096	0.002	320	1.38	0.488	0.107	0.017
OTS-2C	Kr	80	0.065	0.014	0.006	0.004	40	0.726	0.242	0.041	-0.037
OTS-2C	Kw	160	0.187	0.166	0.12	0.075	640	0.967	0.405	0.136	0.041
OTS-3A	Kt	320	1.204	0.523	0.203	0.083	20	0.129	0.052	0.019	0
OTS-3A	Kp	1280	1.346	0.455	0.145	0.054	320	0.222	0.051	0.011	-0.002
OTS-3A	G	2560	1.634	0.641	0.32	0.176	640	0.798	0.303	0.095	0.024
OTS-3A	Kr	320	0.895	0.236	0.066	0.022	40	0.35	0.126	0.053	0.018
OTS-3A	Kw	10240	0.848	0.768	0.606	0.414	640	0.76	0.491	0.252	0.112
OTS-3C	Kt	640	1.136	0.41	0.147	0.05	40	0.399	0.165	0.074	0.03
OTS-3C	Kp	640	1.234	0.42	0.163	0.061	640	0.577	0.233	0.088	0.032
OTS-3C	G	1280	1.079	0.449	0.218	0.126	1280	1.279	0.772	0.374	0.458
OTS-3C	Kr	1280	0.966	0.322	0.107	0.039	80	0.868	0.402	0.156	0.062
OTS-3C	Kw	10240	0.742	0.429	0.295	0.256	2560	1.188	1.007	0.676	0.429
OTS-4A	Kt	80	0.308	0.115	0.059	0.029	20	0.003	-0.006	0.006	-0.006
OTS-4A	Kp	80	0.274	0.086	0.036	0.022	20	0.012	-0.016	-0.008	-0.008
OTS-4A	G	160	0.357	0.147	0.062	0.026	20	-0.01	-0.022	-0.012	-0.007
OTS-4A	Kr	80	0.182	0.044	0.015	0.008	20	0.096	0.022	0.004	-0.002
OTS-4A	Kw	640	1.062	1.025	0.917	0.716	40	-0.008	-0.018	-0.007	0.006
OTS-4C	Kt	320	0.519	0.204	0.08	0.016	40	0.242	0.086	0.034	0.008
OTS-4C	Kp	640	0.384	0.153	0.061	0.021	40	0.305	0.108	0.037	0.009
OTS-4C	G	640	0.504	0.245	0.113	0.052	160	0.252	0.091	0.028	0.008
OTS-4C	Kr	160	0.267	0.089	0.031	0.01	40	0.591	0.242	0.079	0.022
OTS-4C	Kw	10240	1.057	0.958	0.964	0.996	1280	0.48	0.194	0.065	0.024
OTS-5A	Kt	160	1.712	0.73	0.326	0.127	20	0.185	0.111	0.041	0.012
OTS-5A	Kp	160	1.538	0.625	0.227	0.077	80	0.253	0.148	0.063	0.02
OTS-5A	G	320	1.493	0.527	0.19	0.063	640	1.898	1.416	0.838	0.337
OTS-5A	Kr	80	1.284	0.32	0.102	0.033	80	0.22	0.128	0.054	0.027
OTS-5A	Kw	160	0.47	0.234	0.1	0.048	1280	0.563	0.403	0.202	0.082
OTS-5C	Kt	40	2.011	0.977	0.389	0.111	40	0.247	0.137	0.058	0.022
OTS-5C	Kp	640	2.228	1.062	0.443	0.135	160	0.442	0.202	0.081	0.022
OTS-5C	G	1280	2.192	1.137	0.478	0.159	640	2.278	1.785	0.989	0.429
OTS-5C	Kr	40	2.218	0.867	0.332	0.117	40	0.606	0.271	0.109	0.031
OTS-5C	Kw	2560	0.731	0.384	0.215	0.103	1280	1.282	0.681	0.338	0.134

OTS-6A	Kt	640	1.494	0.91	0.365	0.137	640	0.713	0.37	0.117	0.028
OTS-6A	Kp	320	1.522	0.802	0.323	0.106	320	1.291	0.531	0.167	0.041
OTS-6A	G	320	2.212	0.94	0.327	0.095	160	0.547	0.239	0.068	0.017
OTS-6A	Kr	1280	1.689	0.706	0.248	0.069	1280	0.303	0.088	0.02	0.002
OTS-6A	Kw	40	0.159	0.026	0.001	0.001	40	0.158	0.108	0.047	0.009
OTS-6C	Kt	5120	2.023	1.332	0.668	0.24	5120	1.386	0.648	0.257	0.109
OTS-6C	Kp	2560	1.904	1.225	0.578	0.203	5120	1.904	0.927	0.342	0.125
OTS-6C	G	2560	2.676	1.61	0.693	0.226	2560	0.875	0.387	0.13	0.047
OTS-6C	Kr	10240	2.112	1.207	0.58	0.192	5120	0.854	0.293	0.079	0.032
OTS-6C	Kw	320	0.356	0.093	0.027	0.003	640	0.186	0.06	0.013	0.008
OTS-7A	Kt	640	2.252	1.571	0.811	0.353	40	0.32	0.224	0.139	0.045
OTS-7A	Kp	1280	1.921	1.145	0.508	0.206	80	0.2	0.061	0.012	-0.009
OTS-7A	G	5120	1.966	1.195	0.586	0.268	160	0.325	0.136	0.044	0.007
OTS-7A	Kr	640	1.873	1.042	0.443	0.163	40	0.648	0.308	0.108	0.019
OTS-7A	Kw	20480	1.83	1.465	0.951	0.617	1280	0.781	0.385	0.171	0.064
OTS-7C	Kt	320	1.609	0.668	0.226	0.079	20	0.293	0.182	0.074	0.029
OTS-7C	Kp	320	0.962	0.376	0.136	0.05	80	0.171	0.055	0.016	0.006
OTS-7C	G	2560	1.236	0.626	0.27	0.101	320	0.236	0.089	0.037	0.016
OTS-7C	Kr	160	0.719	0.293	0.094	0.036	40	0.649	0.235	0.078	0.031
OTS-7C	Kw	10240	1.084	0.743	0.476	0.306	2560	0.93	0.425	0.179	0.072
OTS-8A	Kt	20	0.061	0.023	0.013	0.011	20	0.074	0.021	-0.005	-0.022
OTS-8A	Kp	20	0.137	0.034	0.005	0.003	20	-0.001	-0.019	-0.02	-0.026
OTS-8A	G	20	0.249	0.06	0.014	0.003	20	-0.004	-0.022	-0.021	-0.023
OTS-8A	Kr	20	0.059	0.021	0.005	0.002	20	0.076	0.039	0.004	-0.016
OTS-8A	Kw	160	0.487	0.178	0.054	0.018	20	0.013	-0.012	-0.017	-0.02
OTS-8C	Kt	160	0.843	0.304	0.121	0.043	40	0.465	0.272	0.123	0.061
OTS-8C	Kp	320	0.76	0.272	0.089	0.028	80	0.117	0.025	0.003	0.012
OTS-8C	G	640	1.202	0.649	0.3	0.114	320	0.825	0.314	0.093	0.042
OTS-8C	Kr	40	0.521	0.171	0.053	0.024	40	0.221	0.096	0.044	0.024
OTS-8C	Kw	10240	1.665	1.332	0.98	0.709	640	0.43	0.217	0.09	0.046
OTS-9A	Kt	20480	1.292	0.724	0.439	0.277	81920	3.63	3.415	2.825	1.854
OTS-9A	Kp	20480	2.014	1.54	0.937	0.523	40960	3.758	3.51	2.921	1.843
OTS-9A	G	20480	1.524	0.847	0.381	0.18	40960	5.479	4.032	3.354	2.152
OTS-9A	Kr	20480	1.406	0.883	0.449	0.216	40960	3.96	3.98	3.072	1.951
OTS-9A	Kw	20480	1.494	0.682	0.241	0.083	640	0.445	0.189	0.063	0.025
OTS-9C	Kt	20480	0.999	0.499	0.355	0.254	20480	3.588	3.359	2.842	2.033
OTS-9C	Kp	20480	1.804	1.441	1.04	0.719	20480	3.92	3.342	2.687	1.805
OTS-9C	G	20480	0.941	0.563	0.327	0.202	10240	4.205	4.179	3.034	2.053
OTS-9C	Kr	20480	0.951	0.634	0.409	0.264	20480	4.206	3.826	3.041	2.051
OTS-9C	Kw	20480	1.047	0.489	0.194	0.075	320	0.844	0.484	0.243	0.07
OTS-12A	Kt	40	0.066	0.025	0.01	0.008	20	0.653	0.252	0.117	0.057
OTS-12A	Kp	320	0.036	0.012	0.003	0.002	20	0.104	0.031	0.019	0.019
OTS-12A	G	320	0.418	0.128	0.041	0.013	20	0.027	-0.017	-0.001	0.006
OTS-12A	Kr	80	0.065	0.019	0.006	0.002	20	1.271	0.473	0.221	0.126
OTS-12A	Kw	160	0.519	0.23	0.102	0.036	20	0.006	-0.03	-0.011	0.002
OTS-12C	Kt	320	0.222	0.089	0.041	0.024	80	1.135	0.502	0.208	0.08
OTS-12C	Kp	640	0.101	0.04	0.022	0.013	320	0.219	0.087	0.031	0.006
OTS-12C	G	2560	1.62	1.099	0.526	0.207	320	0.152	0.072	0.024	0.005
OTS-12C	Kr	160	0.155	0.04	0.01	0.002	160	1.299	0.563	0.242	0.093
OTS-12C	Kw	5120	1.08	0.855	0.581	0.328	1280	1.606	1.515	0.857	0.391
OTS-14A	Kt	20	0.029	0.009	0.005	0.004	20	0.027	0.011	0.002	-0.003
OTS-14A	Kp	20	0.058	0.012	0.004	0.003	20	0.018	0.004	0	-0.003
OTS-14A	G	20	0.041	0.011	0.005	0.002	20	0.02	0.004	-0.001	-0.001
OTS-14A	Kr	20	0.027	0.007	0.003	0.001	20	0.098	0.055	0.033	0.008
OTS-14A	Kw	20	0.072	0.016	0.004	0.003	20	0	-0.004	-0.004	-0.003
OTS-14C	Kt	20	0.157	0.044	0.015	0.003	20	0.048	0.016	0.005	0
OTS-14C	Kp	80	0.17	0.043	0.012	0.005	20	0.02	0.004	0.001	-0.001

OTS-14C	G	160	0.245	0.07	0.025	0.009	40	0.028	0.004	0	-0.002
OTS-14C	Kr	20	0.05	0.015	0.004	0.003	20	0.114	0.052	0.026	0.007
OTS-14C	Kw	320	0.416	0.251	0.129	0.052	320	0.262	0.106	0.045	0.016
OTS-15A	Kt	2560	0.468	0.291	0.201	0.114	20	-0.007	-0.006	-0.007	-0.004
OTS-15A	Kp	2560	0.598	0.337	0.248	0.114	20	0.103	0.023	0.005	0.001
OTS-15A	G	2560	1.022	0.456	0.357	0.089	20	0.198	0.046	0.015	0.005
OTS-15A	Kr	2560	0.105	0.01	-0.004	-0.001	20	-0.002	-0.005	-0.007	-0.003
OTS-15A	Kw	2560	0.105	0.109	0.033	0.015	20	-0.011	-0.009	-0.008	-0.003
OTS-15C	Kt	640	0.369	0.193	0.117	0.054	20	-0.029	-0.012	0.004	0.002
OTS-15C	Kp	640	0.369	0.203	0.114	0.054	20	-0.02	-0.007	-0.003	0.006
OTS-15C	G	640	0.556	0.263	0.105	0.039	20	0.013	0.005	0	0.004
OTS-15C	Kr	1280	0.057	0.015	0.005	0.004	20	-0.012	-0.01	-0.005	0
OTS-15C	Kw	640	0.056	0.024	0.013	0.005	20	-0.04	-0.016	-0.002	0
OTS-16A	Kt	80	0.256	0.237	0.148	0.061	5120	1.072	1.141	0.757	0.329
OTS-16A	Kp	320	0.692	0.434	0.191	0.073	5120	1.642	1.483	0.906	0.358
OTS-16A	G	320	0.416	0.3	0.131	0.046	5120	1.385	1.188	0.715	0.23
OTS-16A	Kr	320	0.515	0.27	0.112	0.034	2560	0.686	0.496	0.225	0.072
OTS-16A	Kw	20	0.09	0.029	0.011	0.008	20	0.028	0.009	-0.001	-0.003
OTS-16C	Kt	2560	0.558	0.594	0.476	0.232	40960	1.632	1.555	1.39	0.969
OTS-16C	Kp	5120	1.203	1.135	0.71	0.343	20480	1.857	2.076	1.809	1.275
OTS-16C	G	5120	0.702	0.703	0.605	0.309	10240	2.219	1.898	1.565	0.984
OTS-16C	Kr	5120	0.985	0.73	0.449	0.209	10240	1.528	1.116	0.588	0.282
OTS-16C	Kw	320	0.409	0.168	0.071	0.023	80	0.587	0.318	0.128	0.041
OTS-18A	Kt	20	0.24	0.074	0.021	0.011	20	0.313	0.117	0.035	0.004
OTS-18A	Kp	20	0.288	0.067	0.015	0.008	20	0.217	0.058	0.013	-0.001
OTS-18A	G	20	0.283	0.059	0.014	0.007	20	0.078	0.01	-0.006	-0.009
OTS-18A	Kr	20	0.141	0.032	0.005	0.003	20	0.154	0.056	0.012	-0.003
OTS-18A	Kw	20	0.118	0.025	0.005	0.002	20	-0.006	-0.009	-0.014	-0.009
OTS-18C	Kt	1280	1.919	1.271	0.781	0.351	5120	1.996	1.357	0.719	0.312
OTS-18C	Kp	1280	1.927	1.391	0.854	0.396	2560	2.176	1.505	0.711	0.309
OTS-18C	G	640	2.313	1.766	1.014	0.482	1280	2.646	2.212	1.225	0.487
OTS-18C	Kr	2560	2.28	1.473	0.664	0.256	5120	3.021	2.348	1.271	0.533
OTS-18C	Kw	640	0.906	0.582	0.277	0.122	80	0.59	0.229	0.067	0.026
OTS-19A	Kt	320	0.953	0.419	0.175	0.064	160	0.491	0.161	0.05	0.011
OTS-19A	Kp	80	0.871	0.313	0.107	0.034	160	1.225	0.518	0.203	0.062
OTS-19A	G	80	0.757	0.302	0.104	0.036	160	0.324	0.133	0.041	0.01
OTS-19A	Kr	640	2.155	1.078	0.41	0.139	5120	4.171	3.733	3.346	2.789
OTS-19A	Kw	20	0.252	0.079	0.024	0.008	20	0.318	0.1	0.022	0.006
OTS-19C	Kt	160	0.451	0.195	0.083	0.034	40	-0.073	-0.02	-0.01	-0.006
OTS-19C	Kp	40	0.292	0.097	0.03	0.017	40	0.3	0.15	0.059	0.019
OTS-19C	G	40	0.293	0.106	0.034	0.013	40	-0.099	-0.03	-0.014	-0.007
OTS-19C	Kr	320	1.124	0.514	0.181	0.067	1280	3.212	3.301	2.923	2.85
OTS-19C	Kw	20	0.047	0.016	0.006	0.002	20	-0.132	-0.043	-0.016	-0.006
OTS-20A	Kt	2560	2.007	1.61	0.956	0.417	40	0.439	0.203	0.072	0.025
OTS-20A	Kp	2560	2.482	1.842	1.231	0.553	640	0.619	0.278	0.125	0.043
OTS-20A	G	2560	3.321	2.946	1.593	0.718	40	0.455	0.213	0.093	0.041
OTS-20A	Kr	2560	3.458	2.361	1.154	0.419	40	1.05	0.447	0.156	0.053
OTS-20A	Kw	1280	1.829	1.238	0.673	0.26	20	0.162	0.056	0.021	0.006
OTS-20C	Kt	20480	2.152	2.241	2.159	1.494	320	0.917	0.561	0.291	0.129
OTS-20C	Kp	20480	2.725	2.596	2.35	1.868	2560	2.44	1.721	1.102	0.559
OTS-20C	G	20480	2.719	3.089	3.126	2.536	640	3.091	2.184	1.387	0.694
OTS-20C	Kr	20480	2.793	3.187	3.03	2.374	2560	3.694	2.797	1.812	0.866
OTS-20C	Kw	10240	2.013	1.903	1.516	1.074	160	1.016	0.741	0.426	0.185
OTS-21A	Kt	80	0.906	0.343	0.129	0.055	40	0.197	0.122	0.067	0.022
OTS-21A	Kp	80	0.739	0.302	0.123	0.053	320	0.192	0.08	0.036	0.015
OTS-21A	G	320	0.802	0.33	0.181	0.083	640	0.512	0.18	0.076	0.027
OTS-21A	Kr	80	1.372	0.353	0.101	0.032	160	0.436	0.188	0.082	0.03

OTS-21A	Kw	160	0.779	0.644	0.406	0.267	640	0.242	0.14	0.064	0.034
OTS-21C	Kt	80	0.975	0.274	0.087	0.028	40	0.189	0.116	0.074	0.032
OTS-21C	Kp	80	0.768	0.235	0.107	0.035	160	0.101	0.077	0.041	0.015
OTS-21C	G	320	0.633	0.309	0.181	0.051	640	0.202	0.141	0.072	0.041
OTS-21C	Kr	80	0.99	0.258	0.081	0.024	320	0.469	0.2	0.091	0.029
OTS-21C	Kw	640	0.913	0.601	0.418	0.109	640	0.437	0.176	0.073	0.015
OTS-23A	Kt	160	1.06	0.585	0.222	0.094	20	0.241	0.09	0.026	0.005
OTS-23A	Kp	80	1.93	0.849	0.277	0.085	40	0.159	0.055	0.017	0.001
OTS-23A	G	80	1.116	0.359	0.129	0.043	80	0.116	0.034	0.006	-0.001
OTS-23A	Kr	160	1.269	0.49	0.173	0.054	20	0.338	0.15	0.057	0.015
OTS-23A	Kw	320	2.143	0.962	0.337	0.113	20	0.068	0.037	0.008	0
OTS-23C	Kt	5120	1.907	1.397	1.058	0.451	20	0.686	0.555	0.382	0.13
OTS-23C	Kp	2560	2.65	2.139	1.688	0.802	160	0.611	0.511	0.338	0.106
OTS-23C	G	5120	2.509	1.532	0.781	0.524	640	0.529	0.385	0.274	0.081
OTS-23C	Kr	2560	2.051	1.383	0.725	0.352	40	1.077	0.981	0.696	0.258
OTS-23C	Kw	10480	2.892	2.248	1.586	0.933	640	0.637	0.452	0.341	0.106
OTS-24A	Kt	80	2.316	0.937	0.326	0.117	20	0.051	-0.007	-0.009	-0.003
OTS-24A	Kp	160	3.014	1.197	0.368	0.115	20	0.019	-0.015	-0.014	-0.008
OTS-24A	G	160	3.229	1.112	0.351	0.107	20	0.024	-0.014	-0.013	-0.006
OTS-24A	Kr	320	2.362	0.746	0.236	0.074	20	0.055	0.003	-0.006	-0.003
OTS-24A	Kw	20	0.307	0.073	0.022	0.009	20	-0.004	-0.033	-0.02	-0.007
OTS-24C	Kt	81920	2.981	5.062	4.139	5.459	1280	1.229	0.805	0.435	0.226
OTS-24C	Kp	40960	4.182	4.138	4.891	5.417	5120	2.558	1.68	0.887	0.403
OTS-24C	G	81920	4.151	4.6	5.323	4.471	2560	1.691	0.976	0.501	0.321
OTS-24C	Kr	81920	3.139	5.03	5.327	5.412	10240	2.312	1.601	0.862	0.397
OTS-24C	Kw	20480	2.441	2.703	2.224	1.64	640	1.172	0.677	0.333	0.145

2. Normal sera

Serum No*	Serotype of antigens	Serum antibody titer - IgM					Serum antibody titer - IgG				
		Micro IF titer	ELISA value				Micro IF titer	ELISA value			
			x100	x400	x1600	x6400		x100	x400	x1600	x6400
N-1A	Kt	20	-0.138	-0.178	-0.157	-0.129	20	0.049	-0.035	-0.038	-0.031
N-1A	Kp	20	-0.153	-0.196	-0.177	-0.142	20	0.042	-0.043	-0.043	-0.031
N-1A	G	20	-0.152	-0.195	-0.175	-0.142	20	0.041	-0.044	-0.041	-0.03
N-1A	Kr	20	0.173	0.083	0.052	0.023	20	0.136	0.038	0.012	0.007
N-1A	Kw	20	-0.182	-0.223	-0.191	-0.155	20	0.035	-0.05	-0.051	-0.033
N-1C	Kt	20	-0.114	-0.158	-0.134	-0.096	20	0.018	-0.035	-0.026	-0.022
N-1C	Kp	20	-0.156	-0.17	-0.144	-0.096	20	0.018	-0.042	-0.033	-0.022
N-1C	G	20	-0.147	-0.169	-0.145	-0.102	20	0.004	-0.049	-0.032	-0.022
N-1C	Kr	20	0.121	0.039	0.038	0.037	20	0.084	0.029	0.011	0.011
N-1C	Kw	20	-0.187	-0.195	-0.16	-0.108	20	0.003	-0.049	-0.035	-0.025
N-3A	Kt	20	0.051	0.011	0.011	0.014	20	0.034	0.006	-0.004	-0.006
N-3A	Kp	20	0.055	0.011	0.007	0.008	20	0.009	-0.001	-0.005	-0.005
N-3A	G	20	0.048	0.008	0.006	0.006	20	0.01	-0.001	0	-0.002
N-3A	Kr	20	0.032	0.006	0.007	0.003	20	0.039	0.021	0.007	0.001
N-3A	Kw	20	0.021	0.003	0.002	0	20	0.001	-0.002	0	0.001
N-3C	Kt	20	0.069	0.02	0.008	0.01	20	0.019	0.001	0.002	-0.001
N-3C	Kp	20	0.049	0.016	0.005	0.006	20	-0.005	-0.001	0	0.001
N-3C	G	20	0.039	0.014	0.003	0	20	-0.003	-0.002	0.002	-0.001
N-3C	Kr	20	0.031	0.008	0.001	-0.001	20	0.034	0.021	0.008	0.001
N-3C	Kw	20	0.024	0.002	0	-0.003	20	-0.01	-0.001	0.001	0.001
N-4A	Kt	20	-0.032	-0.006	0.009	0.01	20	0.112	0.019	-0.003	-0.004
N-4A	Kp	20	-0.034	-0.005	0.004	0.004	20	0.1	0.011	-0.003	-0.003
N-4A	G	20	-0.013	0.001	0.003	0.002	20	0.125	0.014	0.002	0.002
N-4A	Kr	20	0.013	0.006	0.003	0.002	20	0.056	0.008	0.001	0.002

N-4A	Kw	20	-0.038	-0.005	-0.001	0.002	20	-0.011	-0.007	-0.001	0.003
N-4C	Kt	20	-0.002	0.001	0.003	0.008	20	0.087	0.013	0.008	0.002
N-4C	Kp	20	-0.006	-0.001	-0.001	0.003	20	0.06	0.009	0.006	0.001
N-4C	G	20	0.054	0.018	0.005	0.002	20	0.076	0.015	0.009	0.002
N-4C	Kr	20	0.021	0.004	0	-0.001	20	0.036	0.009	0.005	0.001
N-4C	Kw	20	-0.011	-0.005	-0.001	0.001	20	-0.029	-0.006	-0.001	-0.001
N-5A	Kt	20	0.037	0.021	0.017	0.014	20	0.094	0.018	-0.003	-0.004
N-5A	Kp	20	0.026	0.012	0.007	0.009	20	0.009	-0.002	-0.009	-0.005
N-5A	G	20	0.045	0.012	0.007	0.004	20	-0.018	-0.006	-0.004	-0.001
N-5A	Kr	20	0.024	0.006	0.003	0.001	20	0.084	0.028	0.006	0.004
N-5A	Kw	20	0.006	0.001	0.001	0.005	20	-0.025	-0.012	-0.006	0
N-5C	Kt	20	0.049	0.019	0.009	0.003	20	0.069	0.014	0.008	0.003
N-5C	Kp	20	0.024	0.008	0.001	-0.001	20	-0.027	-0.007	0.004	0.002
N-5C	G	20	0.047	0.009	0	-0.003	20	-0.035	-0.011	0.002	0
N-5C	Kr	20	0.02	0.004	0.001	-0.002	20	0.081	0.029	0.013	0.003
N-5C	Kw	20	0.005	0	-0.002	-0.001	20	-0.05	-0.017	-0.001	-0.002
N-6A	Kt	20	0.035	0.007	-0.003	0.005	20	0.002	0.006	0.004	0.002
N-6A	Kp	20	0.024	0.001	-0.004	0.003	20	-0.004	0.002	0.004	0.001
N-6A	G	20	0.01	-0.004	-0.005	0.001	20	-0.004	0.002	0.002	0.003
N-6A	Kr	20	0.034	0.005	0.001	0.004	20	0.02	0.007	0.003	0.002
N-6A	Kw	20	-0.008	-0.005	0	0.001	20	-0.007	0.002	0.005	0.006
N-6C	Kt	20	-0.029	-0.009	-0.003	0.012	20	0	-0.004	0.003	-0.005
N-6C	Kp	20	-0.033	-0.012	-0.006	0.003	20	-0.002	-0.004	0.002	-0.002
N-6C	G	20	-0.042	-0.011	-0.002	0.002	20	0.001	-0.005	0.003	-0.004
N-6C	Kr	20	0.022	0.007	0.002	0.003	20	0.016	0.002	0.002	-0.003
N-6C	Kw	20	-0.047	-0.012	-0.003	-0.001	20	-0.002	0.001	0.005	0.001
N-8A	Kt	20	0.013	0.013	0.002	0.006	20	0.18	0.081	0.034	0.01
N-8A	Kp	20	0.037	0.005	-0.003	0.002	20	0.063	0.016	0.008	0.001
N-8A	G	20	0.007	-0.001	-0.005	0	20	0.037	0.003	0.002	0.001
N-8A	Kr	20	0.039	0.008	-0.003	-0.002	20	0.15	0.084	0.047	0.018
N-8A	Kw	20	0.005	-0.003	-0.002	-0.002	20	0.032	-0.005	-0.003	-0.001
N-8C	Kt	20	0.059	0.018	0.004	0.002	20	0.121	0.073	0.037	0.014
N-8C	Kp	20	0.022	0.004	0	0.004	20	0.016	0.014	0.009	0.005
N-8C	G	20	0.017	0.001	0	-0.001	20	0.001	0.002	0	-0.001
N-8C	Kr	20	0.041	0.01	0.004	0.002	20	0.124	0.091	0.051	0.023
N-8C	Kw	20	-0.003	-0.001	0	-0.001	20	-0.01	-0.009	-0.004	-0.001
N-9A	Kt	20	-0.012	0.004	0.01	0.011	20	0.569	0.254	0.076	0.018
N-9A	Kp	20	-0.013	0.003	0.005	0.008	20	0.134	0.068	0.016	0.001
N-9A	G	20	-0.011	0.006	0.01	0.003	20	0.057	0.032	0.008	0.003
N-9A	Kr	20	0.03	0.008	0.003	0.001	20	0.194	0.139	0.054	0.018
N-9A	Kw	20	-0.027	-0.006	0	0.003	20	0.018	0.011	0	0
N-9C	Kt	20	0.113	0.029	0.005	0.002	20	0.104	0.054	0.024	-0.004
N-9C	Kp	20	0.022	0.01	-0.001	-0.001	20	0.011	0.006	0.006	-0.01
N-9C	G	20	0.052	0.015	-0.002	-0.003	20	0.008	-0.001	0.001	-0.013
N-9C	Kr	20	0.183	0.052	0.014	0.005	20	0.094	0.058	0.027	-0.005
N-9C	Kw	20	0.008	0.002	-0.004	-0.004	20	-0.004	-0.01	-0.002	-0.014
N-10A	Kt	20	-0.005	0.003	0.009	0.015	20	0.002	-0.006	-0.011	-0.005
N-10A	Kp	20	-0.006	-0.006	0	0.005	20	-0.01	-0.01	-0.012	-0.004
N-10A	G	20	-0.029	-0.008	-0.002	0	20	-0.011	-0.009	-0.008	-0.002
N-10A	Kr	20	0.026	0.001	0	0.002	20	0.017	-0.001	-0.005	0
N-10A	Kw	20	0.023	0.005	0	0.003	20	-0.014	-0.012	-0.006	0
N-10C	Kt	20	0.005	0	0.001	0.006	20	-0.004	-0.001	0.004	-0.003
N-10C	Kp	20	-0.015	-0.014	-0.008	0.001	20	-0.019	-0.007	-0.001	-0.004
N-10C	G	20	-0.027	-0.016	-0.009	-0.002	20	-0.016	-0.009	0	-0.006
N-10C	Kr	20	0.05	0.015	0.007	0.004	20	0.048	0.015	0.006	-0.001
N-10C	Kw	20	0.04	0.001	-0.003	0.002	20	-0.033	-0.011	-0.004	-0.007
N-11A	Kt	20	-0.011	0.002	0.001	0.01	20	0.156	0.102	0.075	0.048

N-11A	Kp	20	-0.02	-0.003	0.001	0.009	20	0.028	0.014	0.012	0.003
N-11A	G	20	-0.021	-0.002	-0.003	0.005	20	0.009	0.004	0	-0.001
N-11A	Kr	20	0.002	0.003	-0.003	0.001	20	0.092	0.067	0.053	0.038
N-11A	Kw	20	-0.026	-0.005	-0.005	0.001	20	0.016	0.002	0	-0.002
N-11C	Kt	20	-0.009	0.001	0.089	0.001	20	0.16	0.103	0.082	0.055
N-11C	Kp	20	-0.017	-0.003	0.003	0.001	20	0.02	0.009	0.008	0.011
N-11C	G	20	-0.018	-0.006	0.001	0.003	20	0.005	-0.004	-0.001	0
N-11C	Kr	20	0.012	0.001	0.001	0	20	0.091	0.069	0.059	0.047
N-11C	Kw	20	-0.027	-0.011	-0.005	-0.005	20	0.014	-0.003	0.002	-0.001
N-12A	Kt	20	0.027	0.011	0.013	0.01	20	0.09	0.069	0.05	0.023
N-12A	Kp	20	0.024	0.012	0.007	0.008	160	0.004	0.004	0.004	-0.003
N-12A	G	20	0.015	0.007	0.005	0.002	20	-0.001	-0.003	-0.003	-0.003
N-12A	Kr	20	0.027	0.007	0.002	0.002	40	0.085	0.054	0.043	0.025
N-12A	Kw	20	0.015	0.005	0.001	0.003	40	-0.015	-0.007	-0.004	-0.006
N-12C	Kt	20	0.029	0.016	0.01	0.008	20	0.105	0.069	0.046	0.022
N-12C	Kp	20	0.024	0.014	0.008	0.007	160	0.006	0.006	0.001	-0.006
N-12C	G	20	0.021	0.004	0.003	0.005	20	0	-0.003	-0.006	-0.007
N-12C	Kr	20	0.025	0.006	0.001	0.005	40	0.085	0.052	0.036	0.017
N-12C	Kw	20	0.012	0.008	0.001	0.001	80	-0.011	-0.006	-0.007	-0.008
N-13A	Kt	20	0.023	0.011	0.009	0.01	20	0.049	0.042	0.022	0.006
N-13A	Kp	20	0.005	0.003	0.003	0	20	-0.027	-0.01	-0.008	-0.004
N-13A	G	20	-0.038	-0.005	-0.002	-0.002	20	-0.048	-0.017	-0.013	-0.007
N-13A	Kr	20	0.016	0.003	0	0.001	20	0.043	0.036	0.025	0.009
N-13A	Kw	20	-0.036	-0.009	-0.002	0	20	-0.052	-0.021	-0.011	-0.005
N-13C	Kt	20	0.016	0.009	0.011	0.006	20	0.053	0.049	0.032	0.007
N-13C	Kp	20	-0.003	0.002	0.004	0.006	20	-0.024	-0.004	-0.002	-0.008
N-13C	G	20	-0.02	-0.004	-0.001	0	20	-0.039	-0.013	-0.004	-0.008
N-13C	Kr	20	0.02	0.006	0.001	0	20	0.046	0.041	0.029	0.008
N-13C	Kw	20	-0.024	-0.003	-0.002	-0.002	20	-0.044	-0.009	-0.005	-0.008
N-14A	Kt	20	0.061	0.015	0.005	0.009	20	0.02	0.002	-0.007	-0.009
N-14A	Kp	20	0.041	0.011	0.004	0.006	20	0.022	0.002	-0.005	-0.01
N-14A	G	20	0.045	0.01	0	0.002	20	0.02	0.004	-0.005	-0.006
N-14A	Kr	20	0.039	0.01	-0.001	0	20	0.012	0	-0.006	-0.009
N-14A	Kw	20	0.004	-0.002	-0.001	-0.002	20	0.017	0	-0.006	-0.007
N-14C	Kt	20	0.053	0.021	0.012	0.013	20	0.054	0.007	-0.002	-0.013
N-14C	Kp	20	0.031	0.012	0.006	0.005	20	0.042	0	-0.005	0.011
N-14C	G	20	0.028	0.005	0.003	0.005	20	0.041	0	-0.002	-0.011
N-14C	Kr	20	0.043	0.013	0.004	0	20	0.047	0.012	-0.002	-0.007
N-14C	Kw	20	-0.001	-0.002	0	-0.003	20	0.044	0.007	-0.001	-0.009
N-15A	Kt	20	0.006	0.003	0.002	0.005	20	0.019	0.004	-0.002	-0.005
N-15A	Kp	20	-0.001	-0.002	0	0.002	20	0.002	0.002	0	-0.005
N-15A	G	20	-0.007	-0.003	-0.001	0.002	20	0.002	0	-0.001	-0.003
N-15A	Kr	20	-0.001	-0.001	-0.001	-0.004	20	0.05	0.026	0.005	-0.002
N-15A	Kw	20	-0.003	-0.003	0.002	-0.004	20	-0.006	-0.006	-0.002	-0.001
N-15C	Kt	20	0.014	0.007	0.001	0.002	20	0.003	-0.002	0	-0.019
N-15C	Kp	20	0.007	0.002	0.002	-0.001	20	-0.011	-0.007	-0.003	-0.015
N-15C	G	20	-0.002	-0.003	0	-0.002	20	-0.022	-0.009	0	-0.016
N-15C	Kr	40	0.012	0.002	0	0	20	0.043	0.025	0.006	-0.015
N-15C	Kw	20	0.005	-0.003	0.001	-0.002	20	-0.018	-0.011	-0.002	-0.016
N-16A	Kt	20	0.078	0.027	0.01	0.005	20	-0.003	-0.007	-0.006	-0.006
N-16A	Kp	20	0.125	0.023	0.004	0.006	20	-0.011	-0.011	-0.008	-0.006
N-16A	G	20	0.099	0.017	0.006	0.002	20	-0.003	-0.009	-0.007	-0.003
N-16A	Kr	20	0.069	0.019	0.006	0	20	0.007	-0.005	-0.003	-0.003
N-16A	Kw	20	0.004	-0.008	-0.003	0.001	20	-0.013	-0.008	-0.005	-0.004
N-16C	Kt	20	0.042	0.013	0.013	0.018	20	-0.001	-0.009	-0.004	-0.01
N-16C	Kp	20	0.026	0.006	0	0.012	20	-0.009	-0.008	-0.005	-0.009
N-16C	G	20	0.026	0.004	0	0.001	20	-0.006	-0.007	-0.004	-0.008

N-16C	Kr	20	0.026	0.006	0	0	20	0.005	-0.006	-0.003	-0.009
N-16C	Kw	20	-0.024	-0.008	-0.003	-0.002	20	-0.007	-0.009	-0.003	-0.007
N-17A	Kt	20	0.043	0.015	0.01	0.009	20	0.041	0.016	-0.002	-0.002
N-17A	Kp	20	0.022	0.007	0.004	0.003	20	0.02	0.003	-0.006	-0.001
N-17A	G	20	0.018	0.01	0.001	0	20	-0.005	-0.004	-0.008	-0.003
N-17A	Kr	20	0.033	0.006	0.002	0.002	20	0.065	0.035	0.008	0
N-17A	Kw	20	0.012	0.002	0.001	0.001	20	-0.015	-0.007	-0.008	-0.001
N-17C	Kt	20	0.033	0.014	0.011	0.024	20	0.033	0.01	0.001	-0.004
N-17C	Kp	20	0.02	0.007	0.006	0.007	20	0.015	0.008	-0.001	-0.007
N-17C	G	40	0.017	0.002	0.005	0.001	20	-0.007	-0.006	-0.003	-0.011
N-17C	Kr	20	0.03	0.008	0.007	-0.002	20	0.058	0.031	0.011	-0.003
N-17C	Kw	20	0.006	-0.002	0.001	0.001	20	-0.016	-0.007	-0.004	-0.007
N-18A	Kt	20	-0.245	-0.166	-0.084	-0.027	20	0.009	0.002	-0.004	-0.008
N-18A	Kp	20	-0.109	-0.146	-0.098	-0.041	20	-0.01	-0.008	-0.007	-0.008
N-18A	G	80	-0.061	-0.148	-0.086	-0.035	20	-0.017	-0.015	-0.01	-0.008
N-18A	Kr	40	-0.141	-0.078	-0.052	-0.021	20	0.043	0.034	0.021	0.005
N-18A	Kw	20	-0.242	-0.188	-0.115	-0.054	20	-0.015	-0.016	-0.01	-0.007
N-18C	Kt	20	-0.103	-0.052	-0.01	0.007	20	0.003	-0.001	0.001	-0.012
N-18C	Kp	20	-0.091	-0.061	-0.028	-0.004	20	-0.015	-0.007	-0.005	-0.013
N-18C	G	20	-0.069	-0.052	-0.024	0	20	-0.012	-0.013	-0.008	-0.013
N-18C	Kr	20	0.008	-0.013	-0.007	0.004	20	0.052	0.036	0.021	-0.006
N-18C	Kw	20	-0.118	-0.075	-0.037	-0.014	20	-0.028	-0.012	-0.006	-0.013
N-19A	Kt	20	0.018	0.016	0.014	0.008	20	0	-0.005	-0.011	-0.009
N-19A	Kp	20	-0.004	0.002	0.002	0.006	20	0.013	-0.001	-0.008	-0.007
N-19A	G	20	-0.007	0.003	0.004	0.003	20	0.018	0.001	-0.008	-0.004
N-19A	Kr	20	0.032	0.011	0.004	0.002	20	0.048	0.018	-0.001	-0.003
N-19A	Kw	20	-0.014	-0.002	0	0.002	20	-0.006	-0.004	-0.008	-0.007
N-19C	Kt	20	-0.02	-0.01	0.004	0.015	20	0.011	-0.002	-0.012	-0.009
N-19C	Kp	20	-0.047	-0.021	-0.008	0.001	20	0.044	0.011	-0.008	-0.005
N-19C	G	20	-0.024	-0.019	-0.009	0	20	0.061	0.013	-0.009	-0.006
N-19C	Kr	20	0.054	0.021	0.002	0.002	20	0.13	0.03	0.007	-0.005
N-19C	Kw	20	-0.055	-0.026	-0.011	-0.005	20	0.006	-0.006	-0.009	-0.008
N-20A	Kt	20	0.003	0.003	0.005	0.004	20	0.029	0.002	-0.003	-0.005
N-20A	Kp	20	0.003	0.002	0.002	0.003	20	-0.009	-0.009	-0.006	-0.005
N-20A	G	20	0.001	-0.001	0.002	0.001	20	0.052	0.005	-0.003	-0.002
N-20A	Kr	20	0.008	0.002	-0.001	0	20	-0.009	-0.001	-0.003	-0.004
N-20A	Kw	20	-0.004	-0.001	-0.001	0.002	20	-0.014	-0.006	-0.003	-0.003
N-20C	Kt	20	-0.019	-0.004	0	0.002	20	-0.007	-0.007	-0.011	-0.004
N-20C	Kp	20	0.008	-0.002	-0.002	0.002	20	-0.022	-0.013	-0.012	-0.005
N-20C	G	20	-0.019	-0.009	-0.004	-0.001	20	0.02	0.001	-0.005	-0.003
N-20C	Kr	20	0.011	0.003	-0.002	0	20	0.016	-0.005	-0.007	-0.004
N-20C	Kw	20	-0.023	-0.011	-0.005	-0.001	20	-0.021	-0.012	-0.008	-0.002

3. Sera of other rickettsiosis

		Serum antibody titer - IgM					Serum antibody titer - IgG				
Serum No*	Serotype of antigens	Micro IF titer	ELISA value				Micro IF titer	ELISA value			
			x100	x400	x1600	x6400		x100	x400	x1600	x6400
SFG-4A	Kt	20	0.063	0.003	0.004	0.005	20	0.106	0.067	0.047	0.026
SFG-4A	Kp	20	0.056	-0.003	-0.001	0.001	20	0.032	0.019	0.012	0.006
SFG-4A	G	20	-0.002	0.001	0.001	0.001	20	0.019	0.009	0.002	0
SFG-4A	Kr	20	0.048	0.018	0.006	0.004	20	0.08	0.059	0.046	0.029
SFG-4A	Kw	20	-0.007	-0.004	-0.002	0.002	20	0.003	0	-0.002	-0.002
SFG-4C	Kt	20	0.014	0.007	0.003	0.004	20	0.093	0.06	0.041	0.023
SFG-4C	Kp	20	0	0.001	0	0.001	20	0.034	0.014	0.009	0.004
SFG-4C	G	20	0.009	0.003	0.001	0	20	0.017	0.003	0.004	0

SFG-4C	Kr	20	0.035	0.012	0.005	0.003	20	0.075	0.052	0.04	0.023
SFG-4C	Kw	20	-0.001	-0.001	0	0	20	0	-0.002	-0.001	-0.002
SFG-5A	Kt	20	0.023	0.008	0.016	0.009	20	0.039	0.01	-0.005	-0.005
SFG-5A	Kp	20	0.019	0.003	0.004	0.001	20	0.021	-0.001	-0.006	-0.006
SFG-5A	G	20	0.013	0.002	0.002	0.001	20	0.017	-0.006	-0.009	-0.006
SFG-5A	Kr	20	0.023	0.008	0.004	0.001	20	0.08	0.033	0.007	-0.002
SFG-5A	Kw	20	0.01	0	0.001	0	20	0.008	-0.008	-0.009	-0.006
SFG-5C	Kt	20	0.302	0.106	0.04	-0.121	20	0.12	0.065	0.037	-0.007
SFG-5C	Kp	20	0.265	0.078	0.028	-0.093	20	0.038	0.023	0.008	-0.02
SFG-5C	G	20	0.31	0.1	0.038	-0.128	20	0.037	0.006	0.002	-0.022
SFG-5C	Kr	20	0.257	0.08	0.03	-0.131	20	0.096	0.067	0.044	-0.004
SFG-5C	Kw	20	0.224	0.042	0.008	-0.129	20	0.013	0.001	-0.001	-0.017
SFG-9A	Kt	20	0.065	0.054	0.011	-0.016	20	0.077	0.06	0.074	0.072
SFG-9A	Kp	20	0.049	0.02	-0.015	0.012	20	0.069	0.058	0.064	0.073
SFG-9A	G	20	0.081	0.062	0.025	0.032	20	0.06	0.05	0.07	0.045
SFG-9A	Kr	20	0.09	0.043	0.019	0.018	20	0.089	0.059	0.046	0.041
SFG-9A	Kw	20	-0.019	0.01	0.016	0.006	20	0.058	0.046	0.042	0.042
SFG-9C	Kt	20	-0.018	0	-0.045	0.008	20	0.116	0.094	0.096	0.043
SFG-9C	Kp	20	0.015	0.003	-0.049	0.006	20	0.097	0.093	0.062	0.043
SFG-9C	G	20	0.074	0.048	-0.026	0.002	20	0.088	0.056	0.049	0.041
SFG-9C	Kr	20	0.04	0.062	0.006	0.001	20	0.082	0.056	0.046	0.042
SFG-9C	Kw	20	-0.035	-0.009	-0.026	-0.001	20	0.052	0.045	0.041	0.042
SFG-10A	Kt	20	0.007	0.007	0.004	0.007	20	0.116	0.038	0.011	0.001
SFG-10A	Kp	20	-0.012	-0.002	-0.002	0.002	20	0.003	0.002	0.001	-0.003
SFG-10A	G	20	-0.01	-0.003	-0.001	0.007	20	-0.003	-0.002	-0.003	-0.002
SFG-10A	Kr	20	0	-0.002	-0.002	-0.002	20	0.046	0.022	0.006	-0.001
SFG-10A	Kw	20	-0.013	-0.006	-0.001	-0.003	20	-0.01	-0.004	-0.003	-0.003
SFG-10C	Kt	20	0.007	0.005	0.006	0	20	0.087	0.029	0.011	0.002
SFG-10C	Kp	20	-0.021	-0.005	-0.001	0.001	20	-0.013	-0.005	0	0
SFG-10C	G	20	-0.01	0.001	0.002	0	20	-0.011	-0.008	-0.002	-0.003
SFG-10C	Kr	20	-0.002	0	-0.001	-0.003	20	0.049	0.021	0.007	0.001
SFG-10C	Kw	20	-0.021	-0.006	-0.002	-0.003	20	-0.027	-0.011	-0.003	-0.002
SFG-11A	Kt	20	-0.046	-0.006	0.002	0	20	-0.11	-0.085	-0.042	-0.021
SFG-11A	Kp	20	-0.054	-0.009	-0.004	-0.003	20	-0.154	-0.1	-0.045	-0.016
SFG-11A	G	20	-0.045	-0.007	-0.004	-0.004	20	-0.193	-0.106	-0.048	-0.021
SFG-11A	Kr	20	-0.02	0.001	-0.002	-0.003	20	-0.095	-0.08	-0.038	-0.014
SFG-11A	Kw	20	-0.066	-0.014	-0.002	-0.004	20	-0.091	-0.066	-0.026	-0.009
SFG-11C	Kt	20	0.089	0.022	0.012	0.003	20	0.013	0.018	0.019	0
SFG-11C	Kp	20	0.104	0.016	0.006	0.002	20	-0.069	-0.03	-0.018	-0.005
SFG-11C	G	20	0.104	0.016	0.005	-0.002	20	-0.087	-0.048	-0.018	-0.014
SFG-11C	Kr	20	0.106	0.019	0.006	0	20	0.038	0.04	0.022	0.013
SFG-11C	Kw	20	-0.048	-0.02	-0.006	-0.005	20	-0.079	-0.04	-0.019	-0.007
SFG-12A	Kt	20	0.133	0.034	0	0.01	20	-0.001	-0.023	0.01	-0.008
SFG-12A	Kp	20	0.125	0.03	-0.009	0.008	20	-0.076	-0.068	-0.012	-0.015
SFG-12A	G	20	0.146	0.03	-0.008	0.004	20	-0.105	-0.083	-0.026	0.002
SFG-12A	Kr	20	0.153	0.033	-0.009	0.004	20	0.035	-0.002	0.033	0.022
SFG-12A	Kw	20	-0.023	-0.002	-0.004	0.003	20	-0.078	-0.064	-0.036	-0.016
SFG-12C	Kt	20	-0.036	-0.021	-0.015	-0.006	20	-0.181	-0.182	-0.064	-0.017
SFG-12C	Kp	20	-0.063	-0.033	-0.021	-0.004	20	-0.23	-0.174	-0.059	-0.019
SFG-12C	G	20	-0.048	-0.031	-0.021	-0.004	20	-0.302	-0.207	-0.072	-0.02
SFG-12C	Kr	20	-0.029	-0.023	-0.005	-0.001	20	-0.139	-0.156	-0.043	-0.018
SFG-12C	Kw	20	-0.07	-0.035	-0.003	0	20	-0.168	-0.125	-0.05	-0.004
SFG-15A	Kt	20	0.081	0.027	0.01	0.009	20	-0.067	-0.096	-0.063	-0.033
SFG-15A	Kp	20	0.077	0.029	0.009	0.004	20	-0.07	-0.108	-0.061	-0.032
SFG-15A	G	20	0.073	0.02	0.007	0.006	20	-0.097	-0.123	-0.069	-0.033
SFG-15A	Kr	20	0.051	0.017	0.004	0.001	20	-0.017	-0.074	-0.049	-0.03
SFG-15A	Kw	20	0.101	0.026	0.008	0.004	20	-0.04	-0.035	-0.032	-0.023

SFG-15C	Kt	20	0.117	0.03	0.012	0	20	-0.101	-0.14	-0.068	-0.032
SFG-15C	Kp	20	0.111	0.034	0.01	0	20	-0.131	-0.131	-0.064	-0.028
SFG-15C	G	20	0.116	0.023	0.006	-0.002	20	-0.145	-0.147	-0.069	-0.028
SFG-15C	Kr	20	0.088	0.017	0.004	-0.002	20	-0.049	-0.098	-0.043	-0.021
SFG-15C	Kw	20	0.147	0.038	0.008	-0.001	20	-0.076	-0.07	-0.029	-0.015
SFG-16A	Kt	20	0.107	0.041	0.019	0.012	20	0.026	-0.007	-0.02	-0.011
SFG-16A	Kp	20	0.104	0.026	0.007	0.002	20	0.005	-0.013	-0.021	-0.011
SFG-16A	G	20	0.083	0.022	0.007	0.004	20	0.004	-0.017	-0.022	-0.011
SFG-16A	Kr	20	0.09	0.026	0.007	0.001	20	0.076	0.027	-0.004	-0.004
SFG-16A	Kw	20	0.075	0.02	0.006	0.005	20	-0.011	-0.014	-0.019	-0.006
SFG-16C	Kt	20	0.104	0.036	0.018	0.002	20	0	0.033	-0.019	-0.008
SFG-16C	Kp	20	0.058	0.014	0.004	-0.002	20	-0.01	-0.021	-0.018	-0.01
SFG-16C	G	20	0.044	0.015	0.003	-0.004	20	-0.024	-0.025	-0.019	-0.012
SFG-16C	Kr	20	0.08	0.021	0.004	-0.005	20	0.058	0.024	0.002	-0.003
SFG-16C	Kw	20	0.043	0.008	0.003	0	20	-0.029	-0.025	-0.017	-0.007
SFG-17A	Kt	20	-0.022	0	0.003	0.005	20	-0.007	0.001	0.001	-0.006
SFG-17A	Kp	20	-0.027	-0.004	-0.003	0.002	20	-0.032	-0.019	-0.01	-0.012
SFG-17A	G	20	-0.023	-0.002	-0.003	0.001	40	-0.012	-0.023	-0.012	-0.011
SFG-17A	Kr	20	-0.005	0.008	0	0	20	0.095	0.052	0.03	0.006
SFG-17A	Kw	20	-0.04	-0.007	-0.002	-0.002	20	-0.052	-0.022	-0.011	-0.007
SFG-17C	Kt	20	0.013	0.012	0.007	0.01	20	0.025	0.006	0.005	0
SFG-17C	Kp	20	-0.012	-0.002	-0.001	0.003	20	-0.011	-0.025	-0.012	-0.005
SFG-17C	G	20	-0.001	0.001	-0.001	-0.001	80	-0.005	-0.027	-0.014	-0.006
SFG-17C	Kr	20	0.022	0.007	0	0.002	20	0.088	0.052	0.032	0.015
SFG-17C	Kw	20	-0.02	-0.004	-0.003	-0.003	20	-0.019	-0.024	-0.01	-0.005
TG-1A	Kt	20	-0.001	-0.007	-0.001	0.007	20	-0.198	-0.13	-0.053	-0.025
TG-1A	Kp	20	-0.018	-0.016	-0.006	-0.001	20	-0.173	-0.136	-0.053	-0.025
TG-1A	G	20	-0.018	-0.015	-0.006	0	20	-0.192	-0.139	-0.058	-0.027
TG-1A	Kr	20	0.019	0	-0.002	0	20	-0.158	-0.09	-0.04	-0.021
TG-1A	Kw	20	-0.025	-0.017	-0.007	-0.001	20	-0.168	-0.103	-0.037	-0.02
TG-1C	Kt	20	-0.007	-0.004	0.003	0.007	20	-0.086	-0.102	-0.044	-0.025
TG-1C	Kp	20	-0.023	-0.01	-0.004	0.001	20	-0.098	-0.077	-0.031	-0.016
TG-1C	G	20	-0.022	-0.01	-0.005	0	20	-0.131	-0.109	-0.043	-0.02
TG-1C	Kr	20	0.011	0	-0.002	0.001	20	-0.048	-0.035	-0.02	-0.005
TG-1C	Kw	20	-0.03	-0.012	-0.002	-0.002	20	-0.075	-0.078	-0.024	-0.012
TG-2A	Kt	20	0.023	0.004	0.003	0.01	20	0.004	0.002	0.02	0.018
TG-2A	Kp	20	0.007	-0.003	0.049	0.029	20	-0.147	-0.067	-0.018	0.009
TG-2A	G	20	0.014	0	0.025	0.011	20	-0.16	-0.083	-0.013	0.014
TG-2A	Kr	20	0.029	0.007	0.032	0.027	20	-0.115	-0.034	0.063	0.026
TG-2A	Kw	20	0.002	-0.002	0.01	0.016	20	-0.12	-0.066	-0.022	-0.008
TG-2C	Kt	20	-0.043	-0.053	-0.015	0.003	20	-0.029	0.015	0.057	0.011
TG-2C	Kp	20	-0.013	-0.04	-0.036	-0.009	20	-0.065	0.005	0.018	-0.004
TG-2C	G	40	-0.006	-0.036	-0.012	-0.017	20	-0.111	-0.042	0.017	-0.012
TG-2C	Kr	20	0.115	0.039	0.017	0.013	40	-0.086	0.076	0.069	0.032
TG-2C	Kw	20	-0.002	-0.033	-0.004	-0.02	20	-0.128	-0.048	-0.024	-0.011
TG-3A	Kt	20	0.18	0.07	0.014	0.009	20	-0.018	0.003	0.003	0.004
TG-3A	Kp	20	0.048	0.081	0.046	0.084	20	-0.067	-0.047	-0.025	-0.01
TG-3A	G	40	-0.003	0.031	0.013	0.049	40	-0.095	-0.057	-0.032	0.054
TG-3A	Kr	20	0.187	0.073	0.025	0.011	20	0.014	0.01	0.01	-0.019
TG-3A	Kw	20	0.17	0.025	-0.001	0	20	0.081	-0.027	-0.021	-0.008
TG-3C	Kt	20	-0.004	-0.002	-0.005	0.001	20	-0.098	0.003	0.06	0.038
TG-3C	Kp	20	0.009	-0.008	-0.001	0	20	-0.158	-0.047	0.003	0.023
TG-3C	G	40	0.011	-0.014	0.017	0.005	40	-0.186	-0.081	-0.016	0.037
TG-3C	Kr	40	0.057	0.025	0.007	0.015	20	-0.079	-0.006	0.034	0.044
TG-3C	Kw	20	-0.01	-0.014	-0.009	-0.002	20	-0.127	-0.052	-0.014	-0.002

*OTS: Scrub tyhpus paient, N: Normal people, SFG: other rickettsiosis patient, TG; murin typhus patient, A: 1st serum, C: 2nd serum

$ Kt: Kato, Kp: Karp, G: Gilliam, Kr: Kuroki and Kw: Kawsaki type of *O. tsutsugamushi*
